# Association between increased epicardial adipose tissue volume and coronary plaque composition

**DOI:** 10.1007/s00380-013-0398-y

**Published:** 2013-08-28

**Authors:** Kennosuke Yamashita, Myong Hwa Yamamoto, Seitarou Ebara, Toshitaka Okabe, Shigeo Saito, Koichi Hoshimoto, Tadayuki Yakushiji, Naoei Isomura, Hiroshi Araki, Chiaki Obara, Masahiko Ochiai

**Affiliations:** Division of Cardiology and Cardiac Catheterization Laboratories, Showa University Northern Yokohama Hospital, 35-1, Chigasaki-chuo, Tsuzuki, Yokohama, Kanagawa 224-8503 Japan

**Keywords:** Adipose tissue, Coronary artery disease, Percutaneous coronary intervention

## Abstract

To assess the relationship between epicardial adipose tissue volume (EATV) and plaque vulnerability in significant coronary stenosis using a 40-MHz intravascular ultrasound (IVUS) imaging system (iMap-IVUS), we analyzed 130 consecutive patients with coronary stenosis who underwent dual-source computed tomography (CT) and cardiac catheterization. Culprit lesions were imaged by iMap-IVUS before stenting. The iMAP-IVUS system classified coronary plaque components as fibrous, lipid, necrotic, or calcified tissue, based on the radiofrequency spectrum. Epicardial adipose tissue was measured as the tissue ranging from −190 to −30 Hounsfield units. EATV, calculated as the sum of the fat areas on short-axis images, was 85.0 ± 34.0 cm^3^. There was a positive correlation between EATV and the percentage of necrotic plaque tissue (*R* ^2^ = 0.34, *P* < 0.01), while there was a negative correlation between EATV and the percentage of fibrous tissue (*R* ^2^ = 0.24, *P* < 0.01). Multivariate analysis revealed that an increased low-density lipoprotein cholesterol level (*β* = 0.15, *P* = 0.03) and EATV (*β* = 0.14, *P* = 0.02) were independently associated with the percentage of necrotic plaque tissue. An increase in EATV was associated with the development of coronary atherosclerosis and, potentially, with the most dangerous type of plaque.

## Introduction

Epicardial adipose tissue (EAT) and intra-abdominal visceral fat share a common embryological origin, the splanchnopleuric mesoderm [1]. The pericardial adipose tissue derives its blood supply from noncoronary vessels such as the pericardiacophrenic branches of the internal mammary artery, while EAT is supplied by the coronary arteries. It was reported that the EAT volume is larger in the presence of vulnerable plaques, independently of indices of obesity (body mass index (BMI) and visceral adipose tissue) and the coronary artery calcification (CAC) score [2–7]. In addition, vulnerable plaque is related to coronary events [8], and methods of detecting vulnerable plaques have not been established. The iMAP system (Boston Scientific, Natick, MA, USA) is useful for the assessment of plaque composition by using spectral analysis of intravascular ultrasound (IVUS) radiofrequency data [9]. Accordingly, we investigated the relationship between epicardial adipose tissue volume (EATV) and plaque vulnerability in patients with significant coronary stenosis using a 40-MHz IVUS imaging system [10].

## Patients and methods

### Patients and study design

**Fig. 1:**
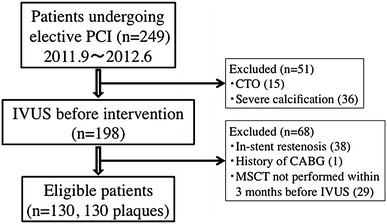
Disposition of the patients. *PCI* percutaneous coronary intervention, *IVUS* intravascular ultrasound, *CTO* chronic total occlusion, *CABG* coronary artery bypass grafting, *MSCT* multislice-row computed tomography

Data were reviewed for 249 consecutive patients with de novo coronary artery lesions who underwent elective percutaneous coronary intervention (PCI) at our institution during the period from September 2011 to June 2012. We selected the most stenotic lesion per subject, and IVUS was performed for vessels with >50 % diameter stenosis on quantitative coronary angiography (QCA). A total of 119 patients were excluded because of in-stent restenosis (*n* = 38), a history of coronary artery bypass graft surgery (*n* = 1), chronic total occlusion (*n* = 15), severe calcification that the IVUS catheter could not cross (*n* = 36), or no multislice computed tomography (CT) within 3 months (*n* = 29) (Fig. 1). As a result, the present study assessed 130 consecutive patients with stenosis who underwent de novo coronary intervention. The smoking status, medical history, and current cardiovascular medications (for hypertension, dyslipidemia, and diabetes) were assessed by a questionnaire. Height (meters) and weight (kilograms) were measured before coronary angiography to calculate the body mass index, and systolic and diastolic blood pressures were measured while the patient was in the supine position before CT was performed.

### PCI strategy and antiplatelet therapy

PCI was performed according to standard methods via the radial or femoral approach using a 6-F or larger guide catheter to facilitate subsequent QCA. Treatment with oral aspirin was started prior to the procedure. Following sheath insertion, unfractionated heparin was administered as bolus doses of 150 units/kg during the procedure to maintain an activated clotting time (ACT) from 250 to 300 s. The ACT was measured both before and during PCI. PCI was performed if patients had no contraindications, such as intolerance to aspirin or ticlopidine and scheduled noncardiac surgery (among others). Additional antiplatelet therapy with either clopidogrel (75 mg/day after a loading dose of 300 mg) or ticlopidine (200 mg/day) was started in all patients after PCI and was continued for at least 1 year.

### Angiographic analyses

The minimum luminal diameter (MLD), reference diameter, and pre- and postprocedural percent diameter stenosis of the lesion were determined with an automated edge detection system (CASSII; PieMedical, Maastricht, The Netherlands). Images were analyzed by a radiologist [11] who was not involved in the study to avoid bias. The contrast-filled catheter tip was used as the calibration standard. All measurements were performed on cine angiograms recorded after intracoronary administration of nitroglycerin. QCA measurements were obtained on both an in-stent basis (confined to the stented region) and in-segment basis (including the vessel 5 mm proximal and distal to the stent). Acute gain was defined as the difference between the postprocedural and preprocedural MLD, while late loss was defined as the difference between the postprocedural MLD and that obtained at follow-up. The reference diameter was defined as the average of the proximal and distal reference diameters of the target vessel before repeat PCI.

### Intravascular ultrasound examination and analysis

**Fig. 2:**
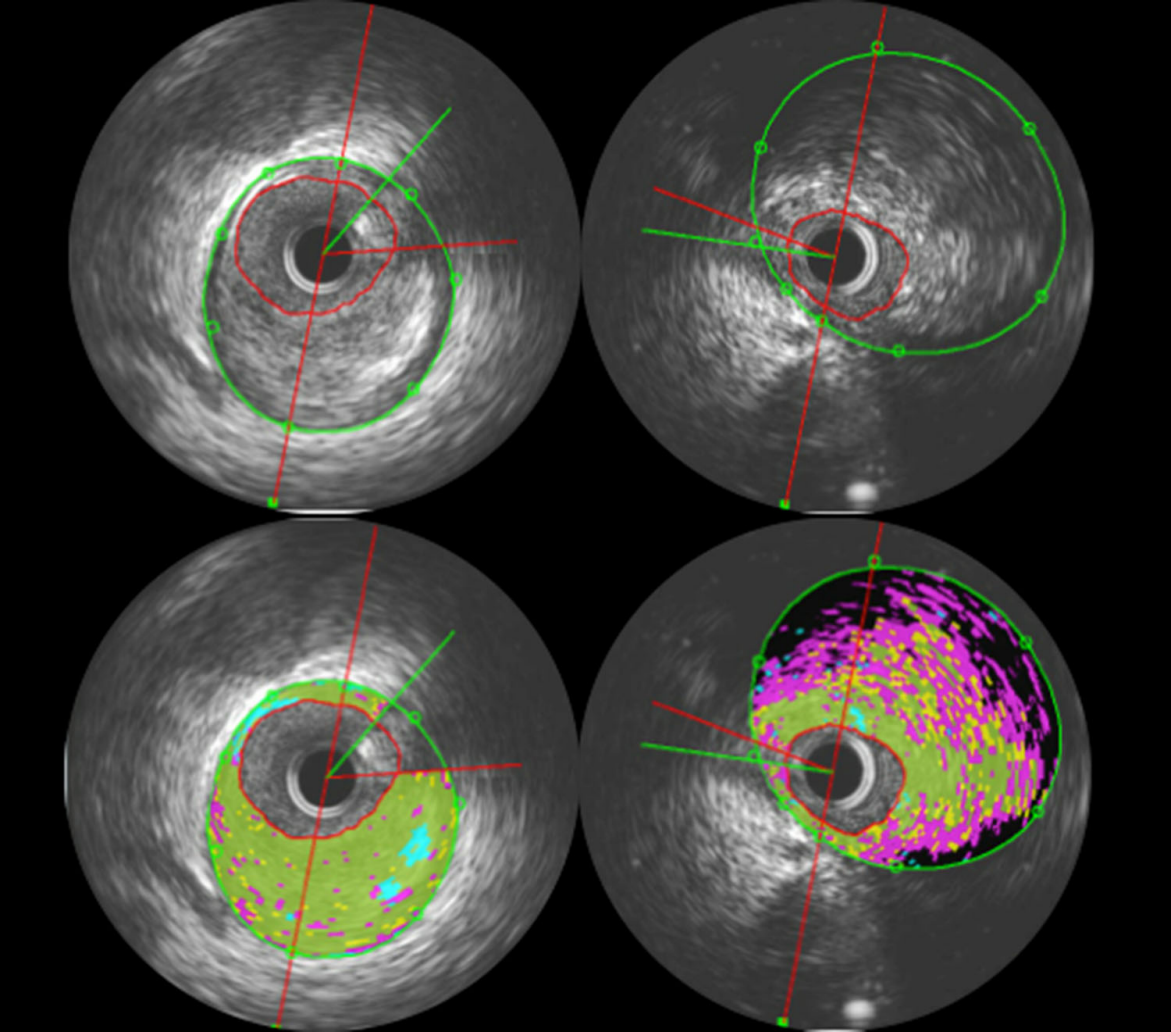
The technique of intravascular ultrasound (IVUS) and iMap classifies the plaque tissue into four color-coded components: green (fibrous), yellow (lipid), pink (necrotic), and blue (calcified). The software used for analysis allows exclusion of the wire shadow from tissue characterization as unknown plaque (*black*) and/or exclusion of the tissue behind calcification

IVUS was performed in all patients before PCI. In brief, after administration of intracoronary nitroglycerin (125–250 μg), a 3.6-F 40-MHz IVUS catheter (Atlantis SR Pro; Boston Scientific) was introduced into the distal coronary artery. This catheter was then withdrawn by an automatic pullback system at a rate of 0.5 mm/s until the coronary ostium was observed. Quantitative volumetric analysis of the IVUS data was performed according to the American College of Cardiology Clinical Expert Consensus Document on Standards for Acquisition, Measurement, and Reporting of IVUS [12]. The vessel and lumen were manually traced at 0.5-mm intervals. Then the luminal volume, vessel volume, and plaque volume (vessel − liminal volume) were computed using Simpson’s method. Plaque burden was calculated as follows: (cross-sectional area of the plaque/cross-sectional area of the vessel) × 100. The minimum luminal cross-sectional area (MLA) was defined as the smallest luminal cross-sectional area inside the lesion. Reference vessel cross-sectional area was defined as the mean of the proximal and distal cross-sectional areas of the reference vessel. The percent stenosis was calculated as (MLA/reference vessel cross-sectional area) × 100, while the remodeling index (RI) was calculated as vessel cross-sectional area at the MLA site/reference vessel cross-sectional area. A 10-mm segment of the culprit lesion (from 5 mm proximal to 5 mm distal to the culprit site) was selected [13] for analysis by iMap-IVUS. In brief, the iMAP system performed spectral analysis of IVUS radiofrequency data. The borders of the vessel and lumen were identified by automatic edge detection and corrected manually when necessary (Fig. 2). Subsequently the iMAP system automatically classified the plaque into four major components, which were fibrous tissue (labeled green), lipid tissue (labeled yellow), necrotic tissue (labeled pink), and calcified tissue (labeled blue), according to a previously reported algorithm [10]. Plaque that was unsuitable for analysis was defined as that affected by acoustic shadowing behind calcification or wire artifacts, and was automatically deleted because it could not be analyzed accurately. The anatomic distribution of plaque in proximal, mid, and distal coronary segments of the left anterior descending artery, left circumflex coronary artery, right coronary artery, and left main artery was also determined.

### CT protocol

A dual-source CT system (Somatom Definition; Siemens Medical Solutions, Forchheim, Germany) was used with the following settings: detector collimation 64 × 0.625 mm, table feed 19.7 mm/s, 0.17 helical pitch (beam pitch), rotation time 280 ms, tube current 370 mA s, and voltage 120 kVp. The scanning time varied from 6 to 8 s. Raw scan data were reconstructed with 75 % of RR wave or the particular optimal phase. A bolus dose of the contrast medium iohexol (Omnipaque; Daiichi-Sankyo Pharmaceutical, Tokyo, Japan) containing 350 mg iodine/ml was injected at 0.6 ml/kg within 9 s. In all patients, a β-blocker (bisoprolol fumarate, 2.5 mg) was administered orally 1 h prior to CT scanning, and nitroglycerin (0.3 mg) was given just before scanning. The reconstructed CT scans were transferred to a workstation for postprocessing (Ziostation; Amin, Tokyo, Japan). CAC was quantified by the Agatston score [14].

### Measurement of EATV

**Fig. 3:**
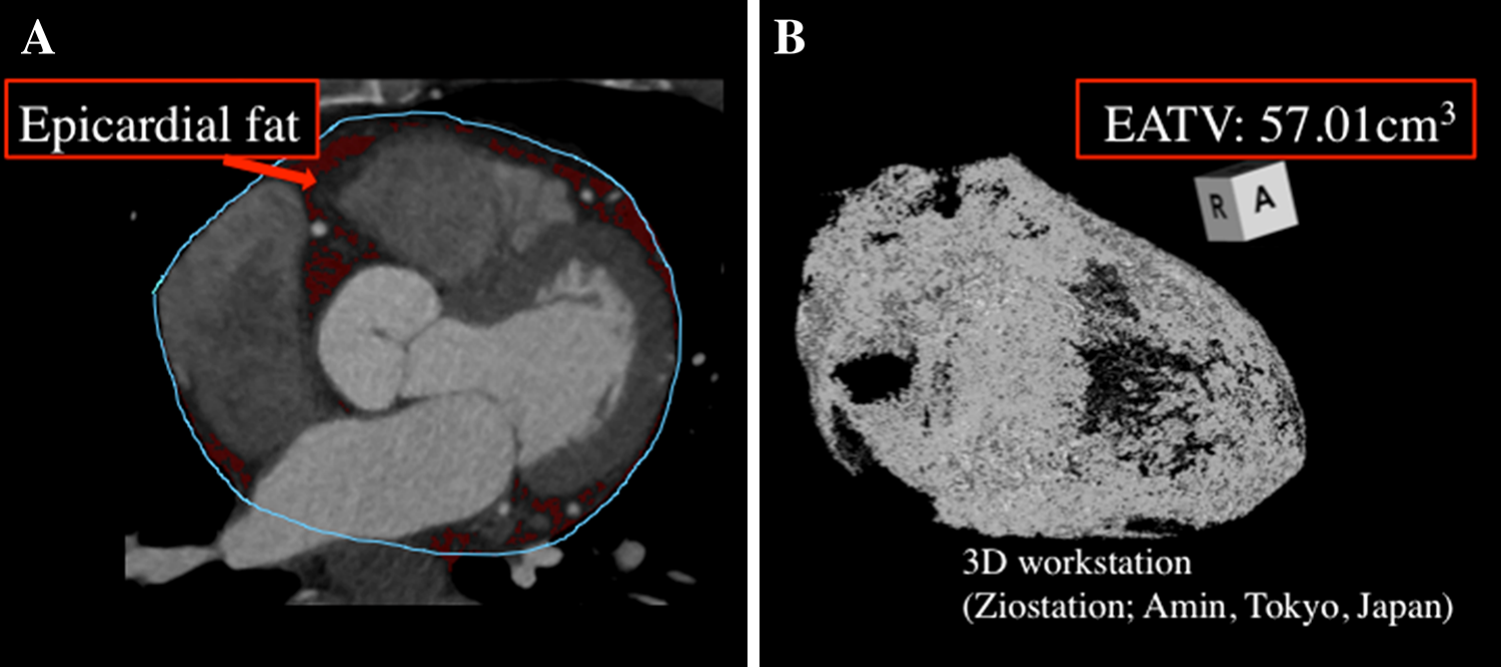
**a** The epicardial adipose tissue area (*red*) was determined by tracing a single region of interest (*blue*) on an axial image. Fat voxels were identified by using a threshold attenuation range of −190 to −30 HU. **b** Epicardial adipose tissue volume (*EATV*) (cm^3^) was automatically calculated as the sum of the fat areas at least 1.0 mm thick for the whole heart

EAT was defined as any adipose tissue located within the pericardial sac. It was identified on contrast-enhanced CT scans as a hypodense rim surrounding the myocardium and limited by the pericardium. Axial slices (0.75 mm thick) of the heart were obtained from the level of the right pulmonary artery to the diaphragm and a predefined image display setting was used (window width of 80 Hounsfield units (HU) and window center of −110 HU) to identify pixels that corresponded to adipose tissue [15]. Readers who were blind to the clinical data then trimmed along the pericardial sac on axial, coronal, and sagittal slices, as well as volume-rendered images (Fig. 3).

### Statistical methods

Data were analyzed with JMP software (SAS Institute, Cary, NC, USA) using analysis of variance (ANOVA). Categorical data are expressed as frequencies and were compared with Pearson’s Chi-square test. Continuous variables are presented as the mean ± standard deviation and were compared using Student’s *t* test or ANOVA. Comparison between coronary plaque tissue composition and EATV was done with univariate linear regression analysis, after which multivariate analysis was performed based on the results of the univariate analysis. In all analyses, a probability (*P*) value of less than 0.05 was considered statistically significant.

### Ethical considerations

Written informed consent was obtained from all subjects, and the ethical committee of our institution approved the study protocol.

## Results

The mean EATV of all patients was 85.0 cm^3^ (range 16.7–186.4 cm^3^). Based on this mean value, patients were divided into a low-EATV group (EATV <85.0 cm^3^, *n* = 65) and a high-EATV group (EATV ≥85.0 cm^3^, *n* = 65).

### High- and low-EATV groups

**Fig. 4:**
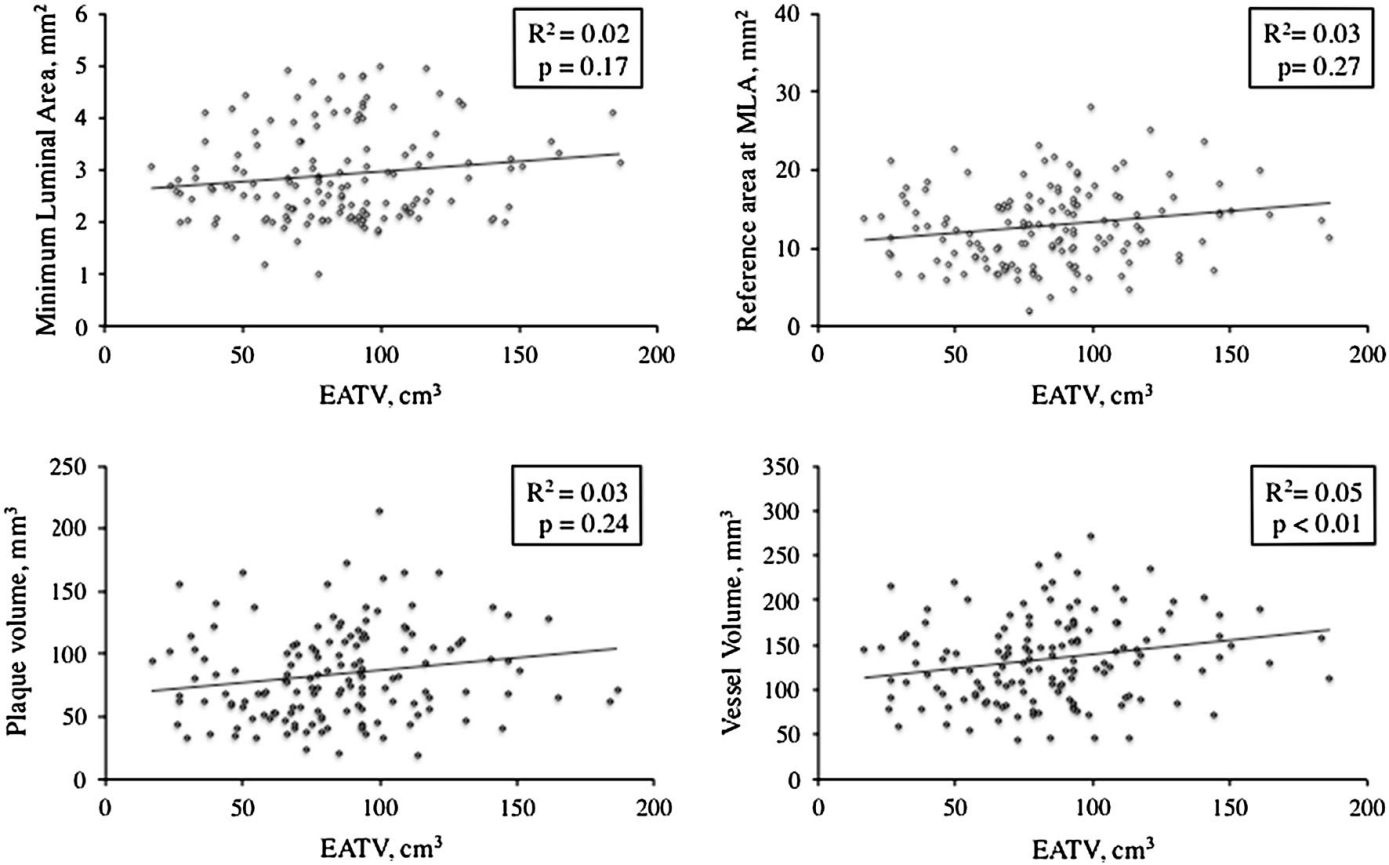
Relationship of epicardial adipose tissue volume (*EATV*) with volumetric intravascular ultrasound parameters. *MLA* minimum luminal cross-sectional area

**Fig. 5:**
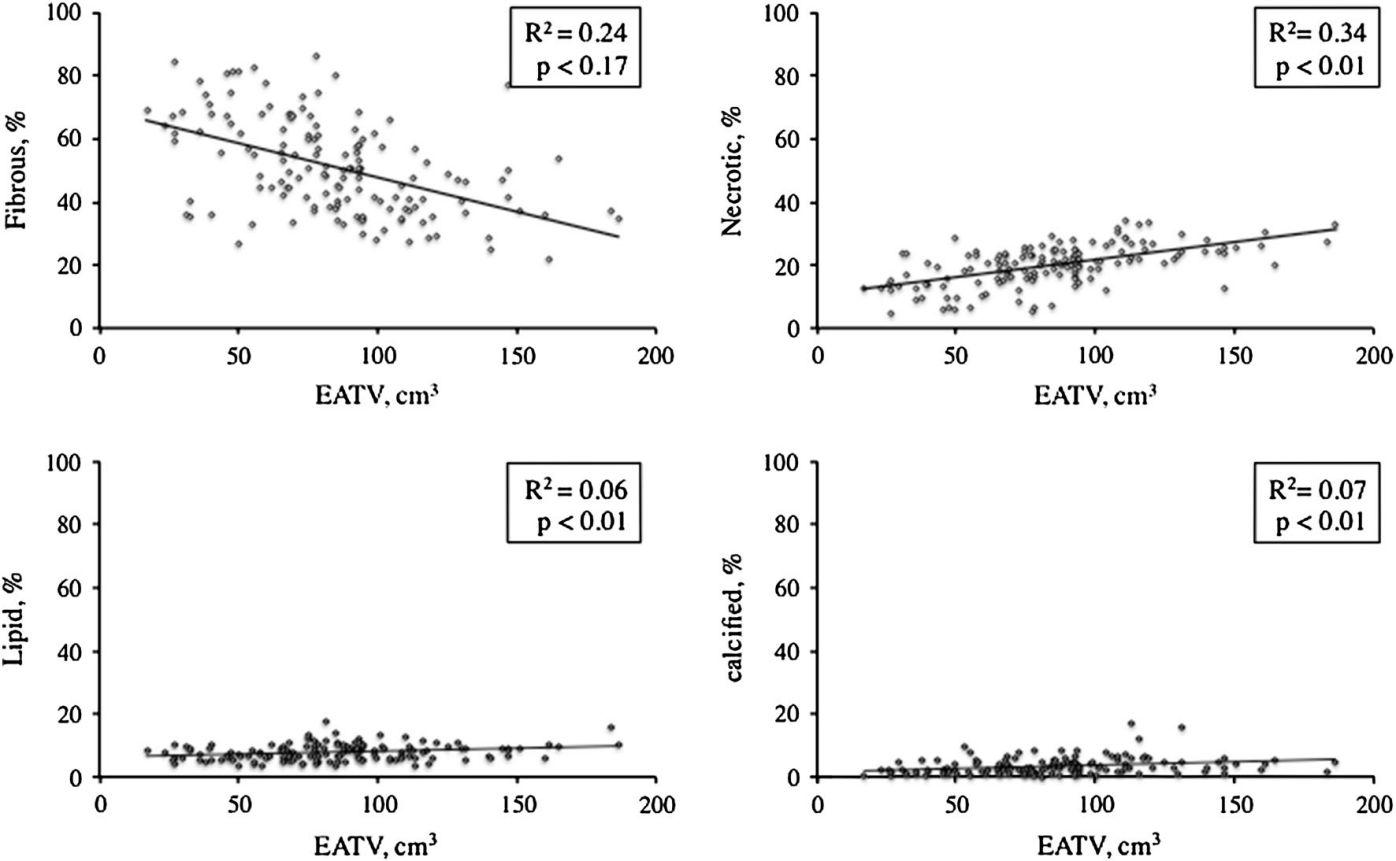
Relationship of epicardial adipose tissue volume (*EATV*) with coronary plaque components

With regard to clinical characteristics, there were no significant differences between the two groups in terms of the age, sex, BMI, and prevalence of diabetes (Table 1). Regarding medications, the percentage of patients using statins was significantly larger in the high-EATV group, accounting for 61.5 % of the high-EATV group versus 38.5 % of the low-EATV group. As shown in Table 2, there were no significant differences between the two groups in terms of angiographic characteristics such as the severity of stenosis, lesion length, and lesion distribution. IVUS data are shown in Table 3. No difference was detected between the two groups with respect to the MLA and lumen volume at the lesions. The reference area at the MLA site was larger in the high-EATV group than in the low-EATV group (76.5 ± 8.3 vs 72.9 ± 10.2, *P* = 0.03), but there was no significant difference in the remodeling index. Also, positive remodeling was more advanced in the high-EATV group (1.08 ± 0.16 vs 0.87 ± 0.14, *P* = 0.04). Plaque volume at the lesion was larger in the high-EATV group than in the low-EATV group. Furthermore, additional analysis of plaque characteristics revealed significantly higher percentages of lipid, necrotic, and calcified tissues in plaque from the high-EATV group. With regard to fibrous tissue, however, no significant difference was detected between the two groups. Subsequently, we assessed the correlation between EATV and MLA (Fig. 4), the vessel diameter at the MLA site, the plaque volume at the lesion site, and the blood vessel volume, but no significant correlations were found among these factors. We also assessed the correlation between EATV and each plaque component (Fig. 5), revealing a significant negative correlation between EATV and fibrous tissue (*R* ^2^ = 0.24, *P* < 0.01), as well as a significant positive correlation between EATV and necrotic tissue (*R* ^2^ = 0.34, *P* < 0.01). However, lipid tissue and calcified tissue showed no significant correlation with EATV. We also performed multivariate analysis of the variables related to necrotic plaque, which is regarded as vulnerable plaque, employing hemoglobin A_1c_ (HbA_1c_), low-density lipoprotein (LDL) cholesterol, high-density lipoprotein (HDL) cholesterol, eicosapentaenoic acid/arachidonic acid (EPA/AA) ratio, highly sensitive C-reactive protein, BMI, and smoking status. As a result, LDL cholesterol (*β* = 0.15, *P* = 0.03) and EATV (*β* = 0.14, *P* = 0.02) were identified as factors predicting plaque with a high percentage of necrotic tissue (Table 4). In addition, we performed multivariate analysis of the variables related to necrotic tissue in high-EATV group. LDL cholesterol (*β* = 0.10, *P* = 0.04) was identified as a factor. However, correlation was very weak.

**Table 1:** Comparison of clinical characteristics

Clinical characteristics	Low-EATV group	High-EATV group	*P* value
No. of patients	65	65	
Age (years)	67.7 ± 10.1	68.0 ± 9.0	0.83
Height (cm)	161.6 ± 9.8	161.1 ± 9.4	0.78
Body weight (kg)	62.1 ± 11.6	61.7 ± 9.8	0.83
BMI (kg/m^2^)	23.6 ± 2.9	23.7 ± 2.8	0.89
Male gender	53 (81.5)	45 (69.2)	0.10
Risk factors			
Family history	24 (36.9)	21 (32.3)	0.58
Hypertension	47 (72.3)	42 (64.6)	0.35
Dyslipidemia	45 (69.2)	41 (63.1)	0.46
Current smoker	18 (27.7)	12 (18.5)	0.21
Diabetes mellitus	19 (29.2)	24 (36.9)	0.35
Total cholesterol (mg/dl)	181.0 ± 37.5	186.7 ± 39.7	0.43
LDL cholesterol (mg/dl)	99.1 ± 30.6	105.7 ± 32.9	0.25
HDL cholesterol (mg/dl)	48.1 ± 12.3	46.0 ± 14.3	0.38
Triglycerides (mg/dl)	162.5 ± 110.7	146.6 ± 100.1	0.41
ApoB/A1 ratio	0.81 ± 0.32	0.87 ± 0.26	0.44
EPA/AA ratio	0.56 ± 0.42	0.58 ± 0.50	0.77
HbA_1c_ (NGSP)	6.22 ± 1.01	6.47 ± 1.25	0.24
Cr (mg/dl)	0.93 ± 0.16	0.95 ± 0.18	0.93
Cystatin C (mg/l)	1.34 ± 0.24	1.39 ± 0.26	0.89
hs-CRP (mg/dl)	0.12 ± 0.18	0.29 ± 0.21	0.19
CAC score	152.3 ± 86.4	188.6 ± 92.2	0.17
Medications			
ARB	23 (35.4)	20 (30.8)	0.58
CCB	29 (44.6)	34 (52.3)	0.38
Statin	25 (38.5)	40 (61.5)	0.01
EPA	6 (9.2)	5 (7.7)	0.75

*AA* arachidonic acid, *Apo* apolipoprotein, *ARB* angiotensin receptor blocker, *BMI* body mass index, *CAC* coronary artery calcification, *CCB* calcium-channel blocker, *Cr* creatinine, *EPA* eicosapentaenoic acid, *HbA*_*1c*_ hemoglobin A_1c_, *HDL* high-density lipoprotein, *hs-CRP* highly sensitive C-reactive protein, *LDL* low-density lipoprotein

**Table 2:** Comparison of angiographic and CT findings

Angiographic findings	Low-EATV group	High-EATV group	*P* value
Pre MLD (mm)	1.11 ± 0.35	1.19 ± 0.41	0.22
Pre diameter stenosis (%)	60.5 ± 12.5	57.4 ± 13.6	0.17
Pre reference diameter (mm)	2.87 ± 0.71	2.92 ± 0.73	0.64
Lesion length (mm)	24.2 ± 13.4	24.4 ± 11.4	0.93
Post MLD (mm)	2.64 ± 0.60	2.68 ± 0.69	0.71
Post diameter stenosis (%)	12.7 ± 7.9	13.5 ± 7.8	0.55
Diseased vessel (%)			0.83
Left main artery	1 (1.5)	1 (1.5)	
Left anterior descending artery			
Proximal	18 (36.9)	22 (49.2)	
Mid	5 (7.7)	8 (12.3)	
Distal	1 (1.5)	2 (3.1)	
Left circumflex artery			
Proximal	8 (8.1)	4 (6.2)	
Mid	4 (6.2)	4 (6.2)	
Distal	5 (7.7)	4 (6.2)	
Right coronary artery			
Proximal	7 (10.8)	8 (12.3)	
Mid	7 (10.8)	8 (12.3)	
Distal	9 (13.8)	4 (6.2)	
CT findings			
Average no. of plaques	2.2 ± 0.6	2.8 ± 0.8	0.11
No. of stenoses			0.49
50–75 %	21 (32.3)	17 (26.2)	
More than 75 %	44 (67.7)	48 (73.8)	
Plaque characteristics			0.93
Noncalcified	40 (61.5)	38 (58.5)	
Mixed	23 (35.4)	25 (38.5)	
Calcified	2 (3.1)	2 (3.1)	
CAC score	64.7 ± 58.4	98.6 ± 66.5	0.16

*MLD* minimum luminal diameter, *CAC* coronary artery calcification

**Table 3:** Comparison of IVUS findings

IVUS findings	Low-EATV group	High-EATV group	*P* value
Vessel volume (mm^3^)	126.6 ± 44.5	144.6 ± 53.7	0.04
Luminal volume (mm^3^)	49.9 ± 19.3	50.6 ± 20.2	0.84
Minimum luminal area (mm^2^)	2.99 ± 1.14	2.98 ± 1.01	0.95
Reference area at MLA (mm^2^)	12.02 ± 4.46	13.80 ± 5.38	0.04
Area stenosis at MLA (mm^2^)	72.9 ± 10.2	76.5 ± 8.3	0.03
Lesion length (mm)	9.9 ± 0.6	9.9 ± 0.7	0.55
Remodeling index	0.87 ± 0.14	1.04 ± 0.16	0.07
Plaque volume (mm^3^)	76.7 ± 32.2	93.9 ± 41.6	0.01
Fibrous (mm^3^)	42.8 ± 16.4	39.2 ± 15.5	0.19
Lipid (mm^3^)	6.0 ± 3.7	7.9 ± 4.2	0.01
Necrotic (mm^3^)	13.6 ± 8.4	22.8 ± 12.2	<0.01
Calcified (mm^3^)	1.9 ± 1.3	3.7 ± 2.3	<0.01
Unknown (mm^3^)	7.5 ± 9.6	14.3 ± 13.2	0.00
Masked (mm^3^)	5.22 ± 4.3	6.6 ± 4.4	0.07
Fibrous (%)	58.6 ± 14.1	43.4 ± 11.0	<0.01
Lipid (%)	7.4 ± 2.4	8.3 ± 2.3	0.03
Necrotic (%)	16.7 ± 5.7	23.7 ± 4.8	<0.01
Calcified (%)	2.8 ± 2.1	4.3 ± 2.7	<0.01

*IVUS* intravascular ultrasound, *MLA* minimum luminal cross-sectional area

**Table 4:** Multivariate analysis between necrotic tissue on iMap-IVUS and several clinical factors in all patients (A) and in the high-EATV group (B)

	Univariate analysis		Multivariate analysis	
	*R* ^2^	*P*	*β*	*P*
(A)				
HbA_1c_	0.18	0.16		
LDL cholesterol	0.38	<0.01	0.15	0.03
HDL cholesterol	0.14	0.83		
EPA/AA ratio	0.08	0.39		
hs-CRP	0.09	0.15		
EATV	0.34	<0.01	0.14	0.02
BMI	0.10	0.43		
Smoking	0.16	0.55		
(B)				
HbA_1c_	0.16	0.61		
LDL cholesterol	0.27	0.04	0.10	0.04
HDL cholesterol	0.06	0.12		
EPA/AA ratio	0.09	0.77		
hs-CRP	0.09	0.15		
BMI	0.18	0.43		
Smoking	0.03	0.22		

*AA* arachidonic acid, *BMI* body mass index, *EPA* eicosapentaenoic acid, *HbA*_*1c*_ hemoglobin A_1c_, *HDL* high-density lipoprotein, *hs-CRP* highly sensitive C-reactive protein, *LDL* low-density lipoprotein

## Discussion

The main results obtained by this study were as follows. First, plaque volume was larger at the lesions in the high-EATV group, indicating more positive remodeling of lesions in this group. Second, there was a negative correlation between EATV and fibrous plaque while there was a positive correlation between EATV and vulnerable plaque containing lipid and necrotic tissue. Third, LDL cholesterol and EATV were identified as independent factors related to necrotic plaque as a result of multivariate analysis. This was the first study to compare the EATV with plaque characteristics and plaque volume at the lesion identified by iMap-IVUS.

### EATV and plaque volume

A relationship between EATV and inflammatory markers has been suggested [16, 17], and EATV was identified as a potential cause of positive remodeling. In the present study, there was no significant difference in MLA between the two groups. Despite the absence of a significant difference between the groups with regard to positive remodeling, the vessel diameter at the lesion tended to be larger in the high-EATV group. The plaque at the site of most stenotic lesion is worthy of discussion. It will be necessary in the future to consider plaque in the nonculprit coronary lesion and the distribution of EAT.

### EATV and plaque vulnerability

The relation between EATV and plaque vulnerability has been assessed using CT data in several previous studies [3–5]. IVUS is superior to CT for characterizing coronary plaque, as described above. In addition, performing iMap-IVUS at 40 MHz is a new technique for more objective and reproducible characterization of plaque components at higher resolution [10]. Our study revealed a positive correlation between EATV and components of vulnerable plaque such as necrotic tissue, while there was a negative correlation between EATV and fibrous tissue. Attenuation accompanied by calcified tissue and artifacts from guide wires are sometimes recognized as necrotic tissue when using the iMap system, whereas the QIVUS software defines these as unknown plaque. Because there was only a small difference in calcified tissue between the two groups compared with that of necrotic tissue, the possibility of the study results being influenced by calcified tissue and wire bias can be ruled out.

### LDL cholesterol and plaque

Although the number of patients receiving statin therapy was significantly greater in the high-EATV group, there was no significant difference between low- and high-EATV groups in terms of LDL cholesterol. However, there was a positive correlation between LDL cholesterol and the amount of necrotic tissue on both univariate and multivariate analyses, hence LDL was reaffirmed as the main causal factor of coronary plaque progression. The LDL-lowering effect of statins is well known to be efficacious for secondary prevention of cardiovascular events [18], and intensive lipid-lowering therapy with strong statins has been frequently reported to prevent cardiovascular events [19–21]. A prospective randomized trial is necessary to firmly establish how intervention is useful for patients with high EATV.

### Limitations

There were some limitations to this study. First, the sample size was relatively small and this was a single-center investigation. Second, only patients who received treatment for coronary artery disease were included in the study. Moreover, they all had stable angina, while those with acute coronary syndromes were excluded (due to problems with accurate diagnosis of thrombus by IVUS), suggesting a possibility of selection bias. Third, follow-up data were not available and epicardial fat was measured only once. Further investigation will be required in the future to assess changes in EATV and plaque volume.

## Conclusion

The present study demonstrated that an increase in EATV was associated with the development of coronary atherosclerosis and, potentially, with the most dangerous type of plaque.
